# IL‐33‐ST2 pathway regulates AECII transdifferentiation by targeting alveolar macrophage in a bronchopulmonary dysplasia mouse model

**DOI:** 10.1111/jcmm.17654

**Published:** 2022-12-27

**Authors:** Yue Zhu, Hui‐ci Yao, Hong‐yan Lu, Xiao‐bo Hao, Su‐qing Xu

**Affiliations:** ^1^ Department of Pediatrics The Affiliated Hospital of Jiangsu University Zhenjiang Jiangsu China

**Keywords:** AECII transdifferentiation, alveolar macrophage, bronchopulmonary dysplasia, IL‐33‐ST2 pathway, lung development

## Abstract

Evidence points to the indispensable function of alveolar macrophages (AMs) in normal lung development and tissue homeostasis. However, the importance of AMs in bronchopulmonary dysplasia (BPD) has not been elucidated. Here, we identified a significant role of abnormal AM proliferation and polarization in alveolar dysplasia during BPD, which is closely related to the activation of the IL‐33‐ST2 pathway. Compared with the control BPD group, AMs depletion partially abolished the epithelialmesenchymal transition process of AECII and alleviated pulmonary differentiation arrest. In addition, IL‐33 or ST2 knockdown has protective effects against lung injury after hyperoxia, which is associated with reduced AM polarization and proliferation. The protective effect disappeared following reconstitution of AMs in injured IL‐33 knockdown mice, and the differentiation of lung epithelium was blocked again. In conclusion, the IL‐33‐ST2 pathway regulates AECII transdifferentiation by targeting AMs proliferation and polarization in BPD, which shows a novel strategy for manipulating the IL‐33–ST2‐AMs axis for the diagnosis and intervention of BPD.

## 1 INTRODUCTION

Bronchopulmonary dysplasia (BPD) is a respiratory disease affecting the survival and prognosis of premature infants.^1^ It was previously confirmed that hyperoxia‐induced Type II alveolar epithelial cells (AECII) to transdifferentiate into fibroblasts through epithelial–mesenchymal transition (EMT) in the lungs of BPD rats, suggesting that the EMT process may affect normal lung development and repair processes of damaged alveoli.^2^

Alveolar macrophages (AMs) are tissue‐resident macrophages of the lung that account for 95% of the lower respiratory tract leucocytes. Macrophages are broadly classified as classically activated macrophages (M1) or alternatively activated macrophages (M2). After co‐culture of lung epithelial cells with M2 macrophages, the expression of α‐SMA (a stromal cell marker) in lung epithelial cells increased, promoting EMT.^3^ Previously, we provided evidence that AMs were elevated in the lungs of BPD mice.^4^ We hypothesized that AM polarization plays a vital role in the repair of alveolar structure and lung abnormalities in BPD. Furthermore, recent studies have suggested that AM activation is associated with IL‐33 levels. There is growing evidence that IL‐33 can induce the M2 phenotype in macrophages during airway inflammation.^5^, ^6^ Therefore, IL‐33‐ST2 plays a crucial role in the functional regulation of macrophages, but its role in BPD remains to be further explored.

Here, we aimed to identify the role of AM in alveolar dysplasia during BPD, and the intrinsic role of the IL‐33‐ST2 pathway in AM proliferation and polarization, by combining macrophage depletion and reconstitution experiments with IL‐33/ST2 knockdown experiments.

## 2 MATERIALS AND METHODS

BPD mice model was established according to previous studies.^7^ For macrophage depletion, BPD mice were orally administered the c‐fms inhibitor GW2580. For IL‐33 and ST2 knockdown, a lentiviral solution with IL‐33‐RNAi‐LV and ST2‐RNAi‐LV was administered intranasally into BPD mice. For IL‐33 administration, BPD mice with ST2 knockdown were injected with rmIL‐33. For AM adoptive transfer, cells were sorted as CD11c^+^F4/80^+^SiglecF^+^cells and injected into BPD mice after IL‐33 knockdown. Flow cytometry analysis, histological analysis, immunofluorescence, Western blot, reverse transcription‐quantitative (RT‐q) PCR, and more detail are described in the Supporting Information. List of abbreviation is described in Table S2.

## 3 RESULTS

### 3.1 Abnormal AECII transdifferentiation in BPD mice

**FIGURE 1:**
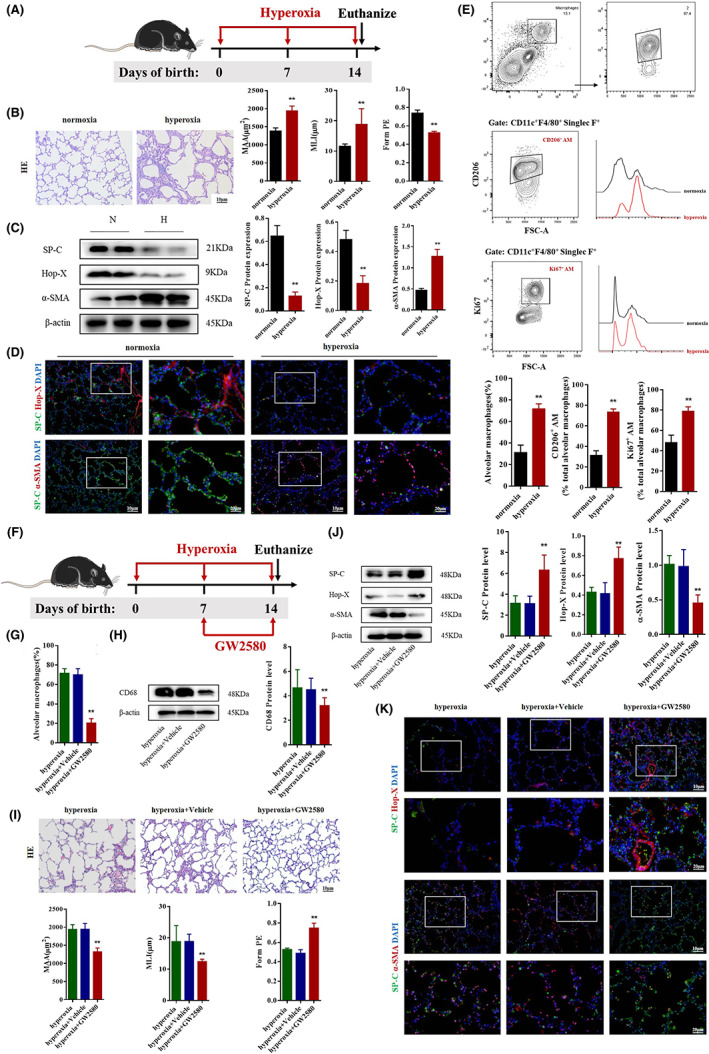
AM played critical roles in AECII transdifferentiation in BPD mice. (A) C57BL/6 newborn mice were treated with normoxia or hyperoxia exposure from birth. Neonatal mice in different groups were sacrificed at Day 14. (B) Representative H&E staining of the neonatal mice lungs in the normoxia and hyperoxia groups. Lung development was assessed by MLI, MAA and form PE; Original magnification, ×200. ** *p* < 0.05 versus normoxia group. (C) Western blot analysis of SP‐C, Hop‐X and α‐SMA in lung tissue of the normoxia and hyperoxia groups. ** *p* < 0.05 versus normoxia group. (D) Representative images of immunofluorescence for SP‐C (green)/ Hop‐X (red) and SP‐C (green)/α‐SMA (red) in the normoxia and hyperoxia groups. Original magnifications (left panel) ×200; square frame magnification (right panel) ×400. (E) Representative results and percentage of AMs (CD11c^+^F4/80^+^Siglec F^+^), CD206^+^ AMs (gate: CD11c^+^F4/80^+^Siglec F^+^) and Ki67^+^ AMs (gate: CD11c^+^F4/80^+^Siglec F^+^) detected by flow cytometry in lung of the normoxia and hyperoxia groups. ** *p* < 0.05 versus normoxia group. (F) During hyperoxia exposure, BPD mice were treated with GW2580 daily for 7 consecutive days before Day 14. Neonatal mice in different groups were sacrificed at day 14. (G) Percentage of AMs (CD11c^+^F4/80^+^Siglec F^+^) detected by flow cytometry in the lungs of the hyperoxia, hyperoxia+Vehicle and hyperoxia+GW2580 groups. ** *p* < 0.05 versus hyperoxia group. (H) Western blot analysis of CD68 in lung of the hyperoxia, hyperoxia+ Vehicle and hyperoxia+GW2580 groups. ** *p* < 0.05 versus hyperoxia group. (I) Representative H&E staining of the neonatal mice lung in hyperoxia group, hyperoxia+Vehicle and hyperoxia+GW2580 groups. Lung development was assessed by MLI, MAA and Form PE. Original magnification ×200. ** *p* < 0.05 versus hyperoxia group. (J) Western blot analysis of SP‐C, Hop‐X and α‐SMA in the lung of different groups. ** *p* < 0.05 versus hyperoxia group. (K) Representative images of immunofluorescence for SP‐C (green)/ Hop‐X (red) and SP‐C (green)/α‐SMA (red). Original magnifications (upper panel) ×200; Square frame magnification (under panel) ×400. Data represented as mean ± SD (*n* = 5).

H&E staining showed that the pulmonary structure of BPD mice was disordered, which was characterized by reduced alveolar number, thickened alveolar septum and simplified structure. MAA and MLI increased significantly, whereas Form PE decreased (Figure 1A,B), like the pathological changes in BPD infants.^8^ Western blot and immunofluorescence showed that the expression of SP‐C (AECII‐specific surface marker) and Hop‐X (AECI‐specific surface marker) were significantly decreased, while α‐SMA was significantly increased in BPD (Figure 1C,D), suggesting that the differentiation of AECII to AECI was decreased and the transdifferentiation of AECII was impaired in BPD.

### 3.2 Abnormal AM proliferation and polarization in BPD mice

The level of AMs, CD206^+^ AM, Ki67^+^ AM and CD68 (a specific macrophage marker) were significantly increased in the lungs of BPD mice compared with normoxia mice (Figure 1E and Figure S1A). Hyperoxia increased the levels of MR, arginase, HO‐1, Fizz1 and IL‐10 in the lung tissues (Figure S1B). These results suggest that hyperoxia induces AM proliferation and polarization.

### 3.3 Macrophage depletion promotes the differentiation of AECII into AECI in BPD mice

BPD mice were orally administered GW2580 on Day 7–14 after birth to deplete monocytes/macrophages (Figure 1F). Flow cytometry and Western blot revealed that AMs decreased significantly after GW2580 treatment (Figure 1G,H). H&E staining showed that after macrophage depletion, the lung tissue structure became regular, MAA and MLI decreased and Form PE increased (Figure 1I). Western blot and immunofluorescence showed that the expression of SP‐C and Hop‐X was upregulated, whereas α‐SMA expression decreased after macrophage depletion (Figure 1J,K).

### 3.4 IL‐33‐ST2 pathway participates in AECII transdifferentiation by targeting AM in BPD mice

The expression of IL‐33 and ST2 was increased in BPD mice and effectively reduced by lentivirus‐mediated siRNAs (Figure S2). Moreover, the levels of AM, Ki67^+^AM, CD206^+^AM and CD68 were reduced after IL‐33 or ST2 knockdown but did not increase with rmIL‐33 stimulation following ST2 knockdown (Figure S3A–E). Moreover, the levels of MR, arginase, HO‐1, Fizz1 and IL‐10 confirmed that IL‐33 induces macrophage polarization through ST2 (Figure S3F).

**FIGURE 2:**
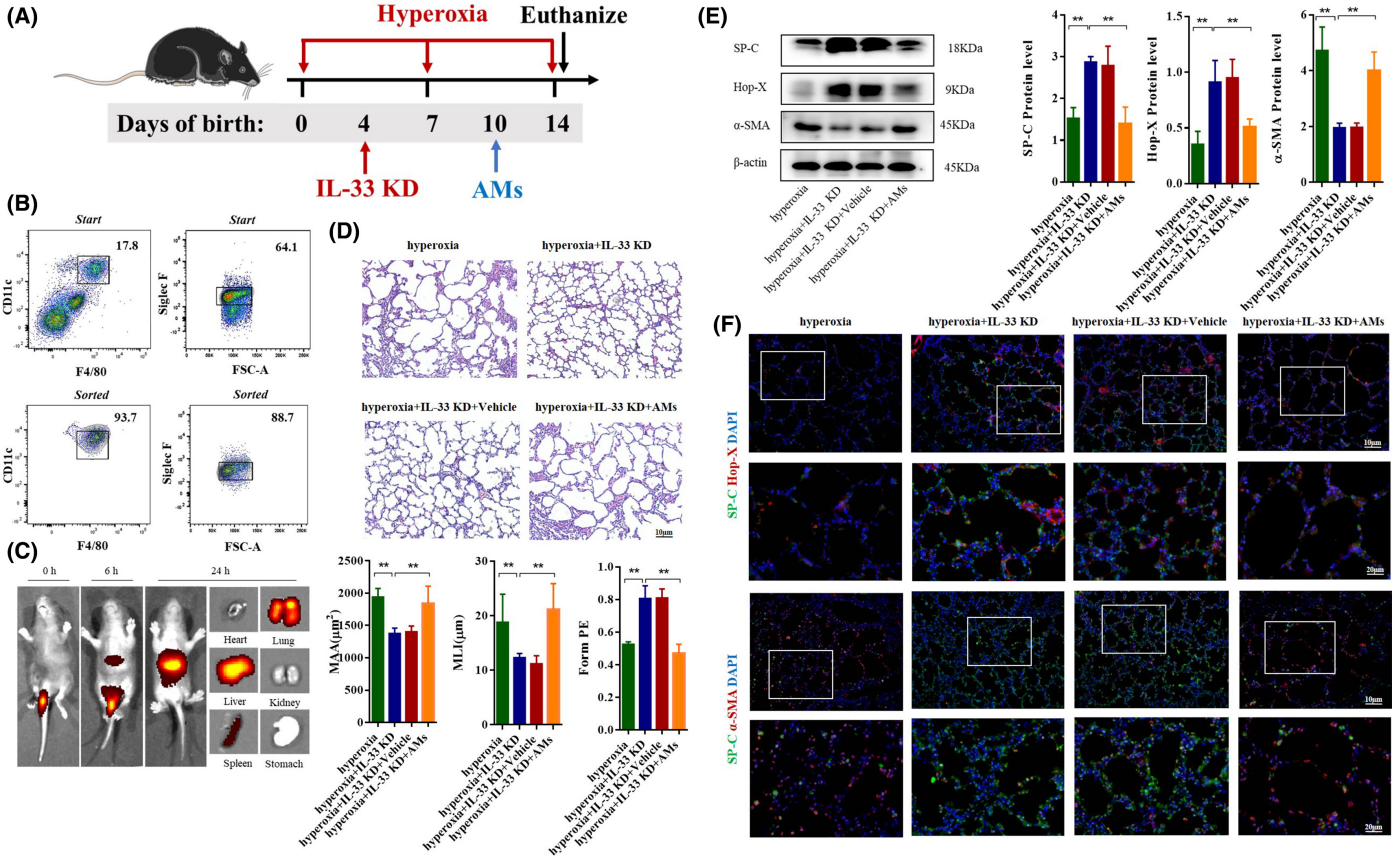
IL‐33 participated in AECII transdifferentiation through AMs proliferation and polarization in BPD mice. (A) During hyperoxia exposure, BPD mice were treated with IL‐33 knockdown at Day 4 and AMs adoptive transfusion at Day 10. Neonatal mice in different groups were sacrificed at day 14. (B) Fluorescence activated cell sorting (FACS); CD11c^+^F4/80^+^Siglec F^+^ lung macrophages sorting and purity analysis. (C) In vivo imaging of Cy7‐stained AMs; Left panel: Whole‐body imaging at 0, 6 and 24 h; Right panel: Ex vivo imaging of heart, lung, liver, spleen, kidney and stomach at 24 h. (D) Representative H&E staining of the neonatal mice lung in the hyperoxia, hyperoxia+IL‐33 knockdown, hyperoxia+IL‐33‐knockdown+Vehicle and hyperoxia+IL‐33‐knockdown+ macrophage groups. Lung development was assessed by MLI, MAA and Form PE. Original magnification ×200. (E) Western blot analysis of SP‐C, Hop‐X and α‐SMA in lung of hyperoxia, hyperoxia+IL‐33‐knockdown, hyperoxia+IL‐33‐knockdown+Vehicle and hyperoxia+IL‐33‐knockdown+ macrophage groups. (F) Representative images of immunofluorescence for SP‐C (green)/Hop‐X (red) and SP‐C (green)/α‐SMA (red); original magnifications (upper panel) ×200; square frame magnification (lower panel) ×400. Data represented as mean ± SD (*n* = 5); ** *p* < 0.05 versus hyperoxia group.

As shown in Figure 2A, CD11c^+^F4/80^+^SiglecF^+^macrophages were transfused into IL‐33 knockdown mice at day 10 (Figure 2A). The macrophages were sorted into CD11c^+^F4/80^+^SiglecF^+^cells and cell purity almost reached 85% (Figure 2B). Cy7‐stained macrophages were mainly concentrated in liver and lungs, and a small number of macrophages was found in the spleen at 24 h after injection (Figure 2C).

After IL‐33 knockdown in BPD mice, the lung structure disorder was improved, the MAA and MLI were decreased, and Form PE was increased. After adoptive transfer, the protective effect of IL‐33 knockdown disappeared, which showed as MAA and MLI increased, and Form PE decreased (Figure 2D). Furthermore, IL‐33 or ST2 knockdown showed that SP‐C and Hop‐X expression were increased, whereas α‐SMA expression was decreased. After ST2 knockdown, the expression of SP‐C, Hop‐X and α‐SMA was not significantly changed with rmIL‐33 stimulation (Figure S4); the levels of SP‐C and Hop‐X were decreased, whereas α‐SMA expression was upregulated in IL‐33 knockdown mice after adoptive transfer of macrophages compared with IL‐33 knockdown mice (Figure 2E). Immunofluorescence staining showed similar results (Figure 2F). These results suggest that the IL‐33‐ST2 pathway participates in the regulation of AECII transdifferentiation in BPD mice and is conducted by AMs.

## 4 DISCUSSION

In this study, we provided evidence that AMs are central to AECII transdifferentiation in BPD, and the intrinsic role of the IL‐33‐ST2 pathway in AM proliferation and polarization, by combining macrophage depletion and reconstitution experiments with IL‐33/ST2 knockdown experiments.

Consistent with previous research,^4^, ^9^, ^10^ this study found that AMs expression increased in BPD mice. Depletion of macrophage partially abolished the EMT process of AECII and alleviated pulmonary differentiation arrest, further supporting that macrophages are regulators of arrested alveolarization in BPD. In addition, we speculated that M2 macrophage may participate in AECII transdifferentiation because: (1) AMs are attached to the surface of AECs^11^; (2) AM‐AEC association and communication play a significant role in lung homeostasis and inflammation^12^; (3) AM polarization can stimulate the EMT process in AECII.^13^ This study found that the variation trends of M2 macrophage and AECII transdifferentiation were highly consistent after treatment with IL‐33 KD, ST2 KD and/or rmIL‐33. Therefore, it is reasonable to infer that M2 macrophage is involved in AECII transdifferentiation in BPD.

Previous studies have revealed that the IL‐33‐ST2 pathway is involved in the macrophage‐mediated repair of multiple organs and tissues.^14^, ^15^ This study provides evidence on the important role of the IL‐33‐ST2 pathway in AM proliferation and polarization. Consistent with decreased M2 macrophage, IL‐33 and ST2 knockdown can protect against hyperoxia‐induced lung injury, which is accompanied by the weakening EMT. Meanwhile, the protective effect of IL‐33 knockdown disappeared following adoptive transfer to IL‐33 knockdown mice, and AECII differentiation was blocked, indicating that the IL‐33‐ST2 pathway participates in BPD by regulating macrophage function.

In conclusion, this report reveals that the IL‐33‐ST2 pathway inhibits the differentiation of AECII into AECI by regulating the excessive proliferation and polarization of AM and promoting EMT in BPD. This study suggests that inhibiting the innate immune axis of IL‐33‐ST2‐macrophage has a strong effect on the treatment of BPD and provides a new strategy to manipulate lung macrophage for treatment options.

## AUTHOR CONTRIBUTIONS

**Yue Zhu:** Data curation (lead); formal analysis (lead); funding acquisition (supporting); methodology (lead); visualization (lead); writing – original draft (lead). **Huici Yao:** Data curation (equal); formal analysis (equal); investigation (supporting); visualization (equal); writing – original draft (equal). **Hongyan Lu:** Conceptualization (lead); funding acquisition (lead); project administration (lead); resources (lead); supervision (lead); writing – review and editing (lead). **Xiaobo Hao:** Data curation (equal); formal analysis (equal); investigation (supporting). **Suqing Xu:** Data curation (equal); formal analysis (supporting).

## CONFLICT OF INTEREST

The authors declare that the research was conducted in the absence of any commercial or financial relationships that could be construed as a potential conflict of interest.

## DATA AVAILABILITY STATEMENT

The datasets generated during and/or analyzed during the current study are available from the corresponding author on reasonable request.

## Supporting information


Appendix S1:
