# Supplementary material for: IL‐33‐ST2 pathway regulates AECII transdifferentiation by targeting alveolar macrophage in a bronchopulmonary dysplasia mouse model
Source: J Cell Mol Med. 2022 Dec 27;27(2):304–8. doi: 10.1111/jcmm.17654 (PMC9843522; doi:10.1111/jcmm.17654)
Supplement: Supplementary file 1 — Appendix S1: [file JCMM-27-304-s001.docx]

IL-33-ST2 pathway regulates AECII transdifferentiation by targeting alveolar macrophage in a bronchopulmonary dysplasia mouse model

Yue Zhu^1^, Hui-ci Yao^1#^, Hong-yan Lu^1*^, Xiao-bo Hao^1^ and Su-qing Xu^1^

^1^Department of Pediatrics, The Affiliated Hospital of Jiangsu University, No.438 Jiefang Road, Zhenjiang, Jiangsu 212001, China

^#^These authors have contributed equally to this work and share first authorship

***Correspondence:**Professor Hong-yan Lu

Department of Pediatrics, The Affiliated Hospital of Jiangsu University, No.438 Jiefang Road, Zhenjiang, Jiangsu 212001, China
E-mail: lhy5154@163.com

**1 MATERIALS AND METHODS**

**1.1 Mice and BPD model**

All the animal experimental protocols were reviewed and approved by the Animal Ethics Committee of Jiangsu University (protocol No. UJS-IACUC-AP-2020030304 and approved on 2020.03.03, Fig. S5). C57BL/6 mice were provided by the Animal Center of Jiangsu University (Zhenjiang, China), and the BPD mice model was established according to previous studies^1^.

**1.2 Macrophage depletion**

C57BL/6 newborn mice were orally administered the c-fms inhibitor GW2580 (cat.no.HY-10917; MCE) at day 7 to deplete monocytes/macrophages at a dose of 150 mg/kg once a day for 7 consecutive days during hyperoxia exposure^2^. Control mice received PBS only, and mice in the different groups were sacrificed at day 14 to evaluate the treatment effects in BPD.

**1.3 IL-33/ST2 treatment**

For IL-33 and ST2 knockdown, small interfering RNAs (siRNAs) were designed to target the nucleotide sequences of mouse IL-33 and ST2 genes. A lentiviral solution containing siRNAs against IL-33 and ST2 and negative control was provided by Shanghai GeneChem (Shanghai GeneChem Co., Ltd). A lentiviral solution with IL-33-RNAi-LV and ST2-RNAi-LV (3×10^8^ TU/mL) was administered intranasally on day 4 during hyperoxia exposure. The mice were exposed to isoflurane for 30 s to achieve mild anesthesia, and the lentiviral solution was administered into nasal passages using a microprojection syringe. Slight pressure was applied to the bottom of the jaw with the thumb to force the mice to inhale solution through the nose. Gauldie et al.^3^ showed that this approach results in a widespread distribution of vectors throughout the lungs. Lung tissue were collected at day 14 and subjected to RT-qPCR or western blot to test the efficiency of IL-33 or ST2 knockdown. For IL-33 administration, BPD mice with ST2 knockdown treatment were injected with rmIL-33 (cat.no.580506; BioLegend) intraperitoneally at day 10, 0.3 mg/day for 4 consecutive days during hyperoxia exposure^4, 5^. Control animals received PBS only.

**1.4 Alveolar macrophage sorting and adoptive transfer**

Macrophages were isolated from lung tissues of BPD mice. Cells were stained and sorted as CD11c^+^F4/80^+^Siglec F^+^ cells using a flow cytometer (BD FACS II; BD). The sorted AMs were incubated with 5 μg/mL Cy7 Dic18 fluorescent cyanine dye (cat.no.HY-D1048; MCE) for 30 min. The cell suspension was centrifuged at 1,000 rpm for 5 min at 4 °C, washed, resuspended, and injected intravenously into BPD mice with IL-33 knockdown treatment (1×10^6^ cells/mouse). Control mice received only PBS. The mice were continuously exposed to hyperoxia, and whole-body imaging was performed using a Spectrum CT machine (PerkinElmer) at 0, 6, and 24 h after lung macrophage reinfusion. BPD mice were sacrificed to harvest the heart, lung, liver, spleen, kidney, and stomach, and ex vivo imaging of the organs was performed.

**1.5 Flow cytometry analysis**

Lung tissues were sectioned into small pieces and 1 mg/mL collagenase A (Roche) was added and digested at 37 °C for 30 min. The digestion solution was filtered through a 70 µm cell strainer (BD) to obtain a single-cell suspension. After red blood cell lysis (Bioscience), the digests were incubated with an Fc-receptor–blocking antibody. Cells were stained for 30 min with FITC anti-CD11c (cat.no.MA5-16877; Invitrogen, Thermo Fisher Scientific), APC anti-F4/80 (cat.no.123116; BioLegend), PE cy7 anti-siglec F (cat.no.25-1702-82; eBioscience, Thermo Fisher Scientific), PE anti-CD206 (cat.no.141706; BioLegend) and BV605 anti-Ki67 (cat.no.652413; BioLegend). Data were acquired using a flow cytometer (BD FACS Canto; BD) and analyzed using Flow Jo.v7 software (BD).

**1.6 Histological analysis**

Paraformaldehyde-fixed lung tissues were dehydrated with alcohol and xylene to prepare 3-μm sections for subsequent experiments. The sections were used for hematoxylin and eosin (H&E) staining for morphometric analysis. The mean linear intercept (MLI, which reflects the mean alveolar diameter), mean alveolar area (MAA, which reflects the average area of alveoli), and Form PE (which reflects the regularity of alveolar shape) provide a simple and accurate assessment of lung development^6, 7^. The images were analyzed with Image Pro-Plus 6.0 (Media Cybernetics).

**1.7 Immunofluorescence**

Immunofluorescence for SP-C (1:100; cat.no.ab270521; Abcam)/Hop-X (1:100; cat.no.sc-398703; Santa Cruz Biotechnology), SP-C/α-SMA (1:100; cat.no.ab240654; Abcam) was performed on the lung tissue sections. Lung tissue sections were blocked with 5% serum and incubated with primary antibodies at 4 °C overnight after deparaffinization and antigen retrieval. After rewarming the next day, the sections were incubated with secondary antibodies (1:1000; cat.no.ab150077, cat.no.150116; Abcam). Immunofluorescence staining of the lung tissues was visualized using an inverted ﬂuorescent microscope.

**1.8 Western Blot**

Total protein was extracted from the lung tissue samples. After electrophoresis and membrane transfer, the PVDF membrane was blocked and incubated with the following primary antibodies at 4 °C overnight: SP-C (1:1000; cat. no.ab270521; Abcam), Hop-X (1:1000; cat.no.sc-398703; Santa Cruz Biotechnology), α-SMA (1:1000; cat.no.ab240654; Abcam), and IL-33 (1:1000; cat.no.MAB3626; R&D Systems). The membranes were washed with TBST and incubated with horseradish peroxidase (HRP)-labeled secondary antibodies (1:5000; FMS-MS01, FMS-Rb01; Fcmacs Biotech) for 1 h at 37 °C. Immunoreactive bands were visualized using Amersham imager600 (Boston). The density of protein content was calculated by LANE 1D software (Sage), and the relative protein expression levels were calculated as target protein/β-actin.

**1.9 Reverse transcription-quantitative (RT-q) PCR**

Total RNA was extracted from the lung tissues using the TRIzol reagent (Invitrogen, Thermo Fisher Scientific) according to the manufacturer's protocols, and the total RNA concentration was determined using a spectrophotometer. In accordance with the instructions of the reverse transcription kit (Thermo Fisher Scientific), the first strand of cDNA was synthesized from the RNA samples. The amplification was performed using a Roche Light Cycler Sequence Detection System (Roche). The amplification program was 95 °C for 30 s, 40 cycles of 95 °C for 30 s, and 60 °C for 34 s. The relative RNA expression levels were quantified using the 2^−△△CT^ method^8^. β-actin was used as the internal control. Primer sequences used in this study are listed in Table S1.

**1.10 Statistical analysis**

All experiments were repeated 5 times (n=5). The results are presented as the mean ± standard deviation (SD). All data were analyzed using SPSS 22.0. Comparisons between two groups were performed using the independent samples *t*-test, while comparisons among multiple groups were performed using one-way analysis of variance (ANOVA) with Tukey’s multiple comparison post hoc test. *P* < 0.05 was considered statistically significant. List of abbreviation is described in Table S2.

**REFERENCES**

1. Warner BB, Stuart LA, Papes RA et al. Functional and pathological effects of prolonged hyperoxia in neonatal mice. *Am J Physiol*. 1998; 275(1):L110-117.

2. Cao Q, Wang Y, Niu Z, et al. Potentiating Tissue-Resident Type 2 Innate Lymphoid Cells by IL-33 to Prevent Renal Ischemia-Reperfusion Injury. *J Am Soc Nephrol*. 2018; 29(3):961-976.

3. Gauldie J, Galt T, Bonniaud P, et al. Transfer of the active form of transforming growth factor-beta 1 gene to newborn rat lung induces changes consistent with bronchopulmonary dysplasia. *Am J Pathol*. 2003; 163(6):2575-2584.

4. Schmitz J, Owyang A, Oldham E, et al. IL-33, an interleukin-1-like cytokine that signals via the IL-1 receptor-related protein ST2 and induces T helper type 2-associated cytokines. *Immunity*. 2005; 23(5):479-490.

5. Besnard AG, Guabiraba R, Niedbala W, et al. IL-33-mediated protection against experimental cerebral malaria is linked to induction of type 2 innate lymphoid cells, M2 macrophages and regulatory T cells. *PLoS Pathog*. 2015; 11(2):e1004607.

6. Crowley G, Kwon S, Caraher EJ, et al. Quantitative lung morphology: semi-automated measurement of mean linear intercept. *BMC Pulm Med*. 2019; 19(1):206.

7. Cox AM, Gao Y, Perl AT, et al. Cumulative effects of neonatal hyperoxia on murine alveolar structure and function. *Pediatr Pulmonol*. 2017; 52(5):616-624.

8. Livak KJ, Schmittgen TD. Analysis of relative gene expres‑ sion data using real‑time quantitative PCR and the 2(‑Delta Delta C(T)) method. *Methods*. 2001; 25(4):402‑408.

**2 SUPPLEMENTARY FIGURE**

*
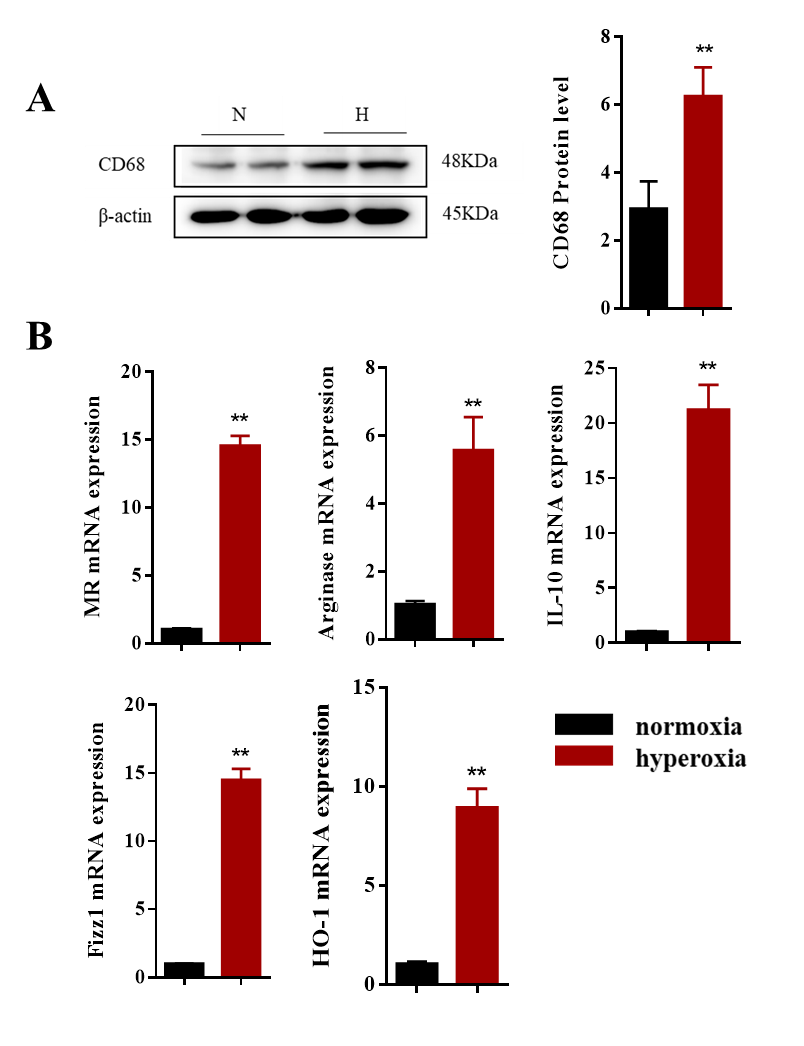
*

**Supplementary Figure 1. Hyperoxia induced abnormal AM proliferation and polarization in BPD mice**

(A) Western blot analysis of CD68 in lung of the normoxia and hyperoxia groups. (B) RT-qPCR analysis of markers of M2 macrophage, including MR, Arginase, HO-1, Fizz1 and IL-10, in lung of the normoxia and hyperoxia groups. Data represented as mean ± SD (n = 5); ** *P* < 0.05 vs hyperoxia group.


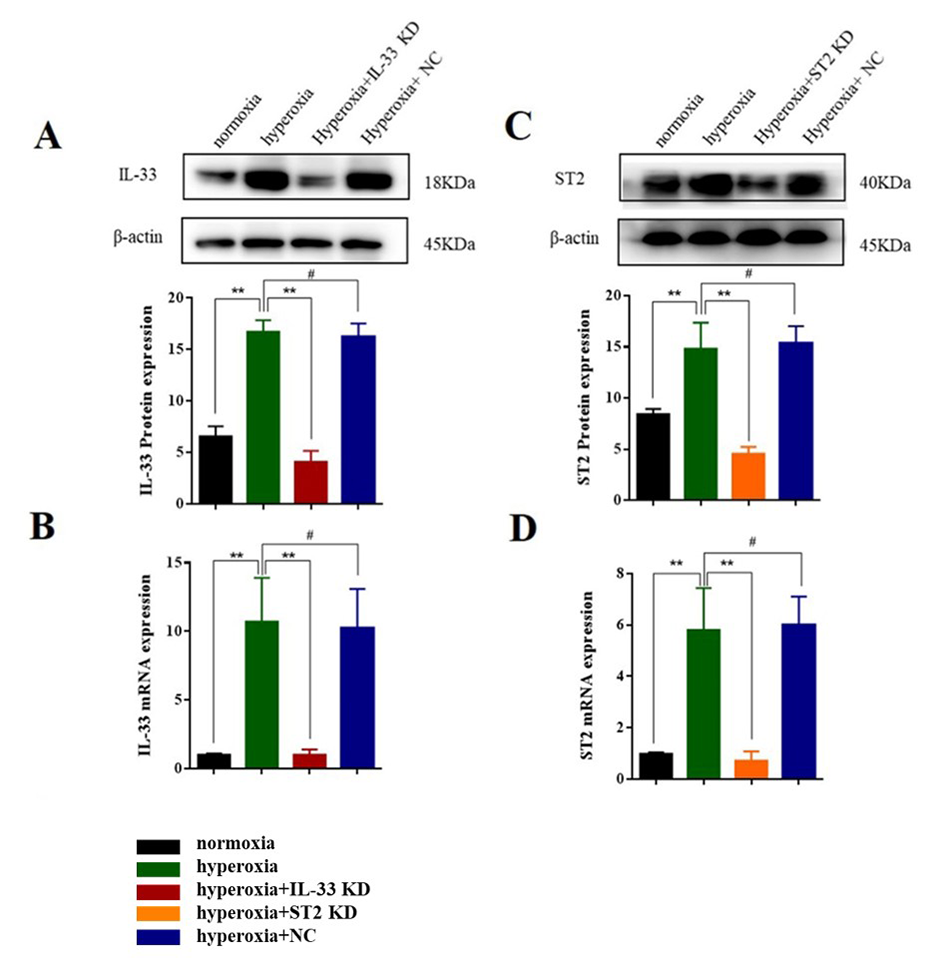


**Supplementary Figure 2. Treatment of IL-33 and ST2 knockdown in lung tissue of BPD mice**

(A) Western blot and (B) RT-qPCR analysis of IL-33 protein and mRNA expression in lung tissues following IL-33 knockdown. (C) Western blot and (D) RT-qPCR analysis of ST2 protein and mRNA expression following ST2 knockdown. Data represented as mean ± SD (n = 5); ** *P* < 0.05 vs hyperoxia group.


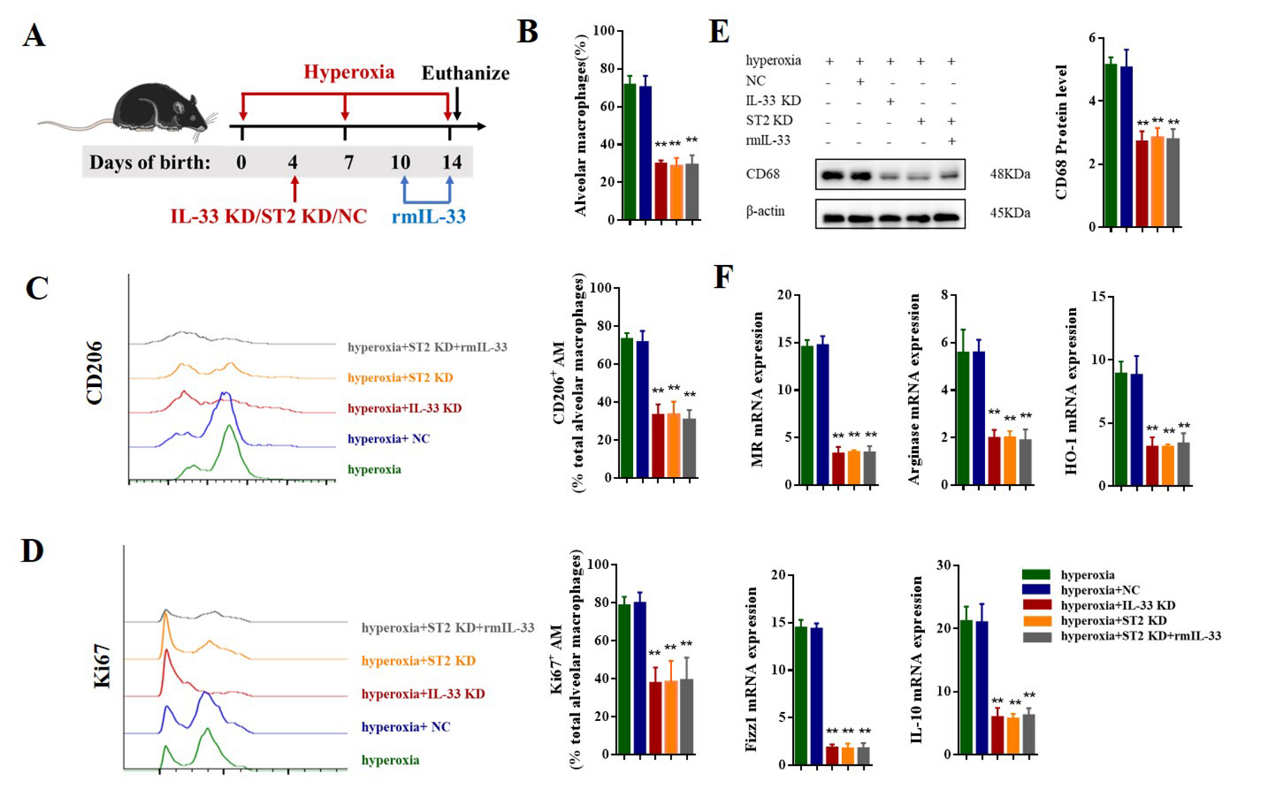


**Supplementary Figure 3. IL-33-ST2 pathway affected AM proliferation and polarization in BPD mice**

(A) During hyperoxia exposure, BPD mice were treated with IL-33 or ST2 knockdown at day 4 and rmIL-33 daily for 4 consecutive days at day 10. Neonatal mice in different groups were sacrificed at day 14. (B) Percentage of AMs (CD11c^+^F4/80^+^Siglec F^+^) detected by flow cytometry in the lungs of different groups. (C) Representative results and percentage of CD206^+^ AMs detected by flow cytometry in lung of different groups. (D) Representative results and percentage of Ki67^+^AMs detected by flow cytometry in the lungs of different groups. (E) Western blot analysis of CD68 in lung of different groups. (F) RT-qPCR analysis of markers of M2 macrophage, including MR, arginase, HO-1, Fizz1 and IL-10, in the lungs of different groups. Data represented as mean ± SD (n = 5); ** *P* < 0.05 vs hyperoxia group.


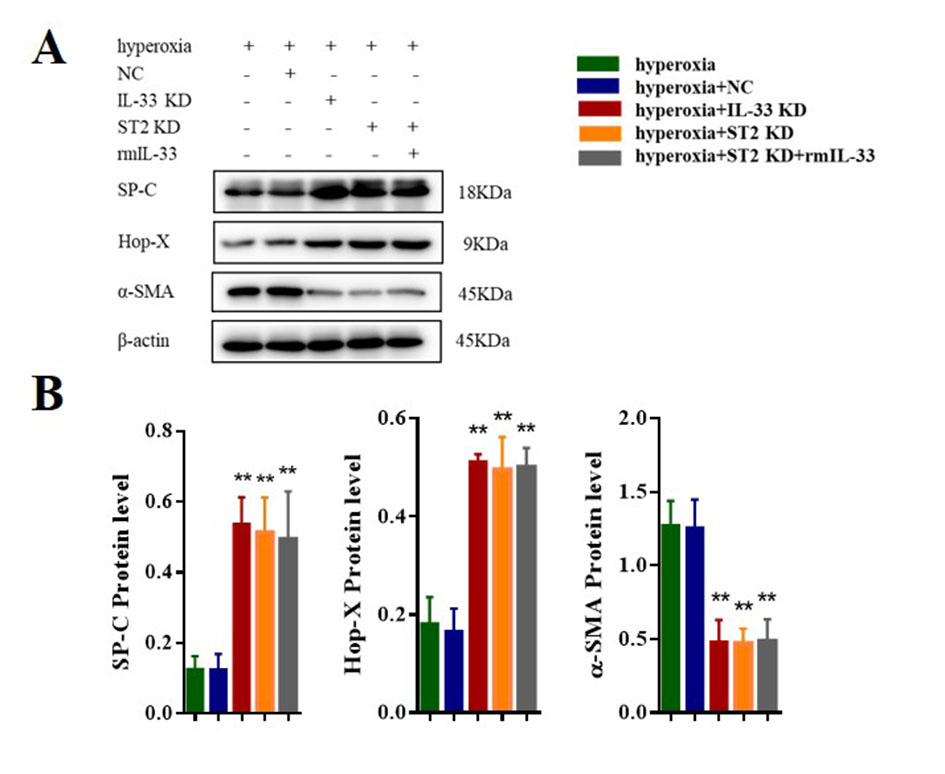


**Supplementary Figure 4. IL-33-ST2 pathway participated in AECII transdifferentiation in BPD mice**

(A) Western blot analysis of SP-C, Hop-X and α-SMA in the lungs of the hyperoxia group, hyperoxia+NC group, hyperoxia+IL-33-knockdown, hyperoxia+ST2-knockdown and hyperoxia+ST2-knockdown+rmIL-33 groups. (B) Quantification of SP-C, Hop-X and α-SMA, protein levels based on densitometry analyses of western blot shown in the panels, respectively. Data represented as mean ± SD (n = 5); ** *P* < 0.05 vs hyperoxia group.


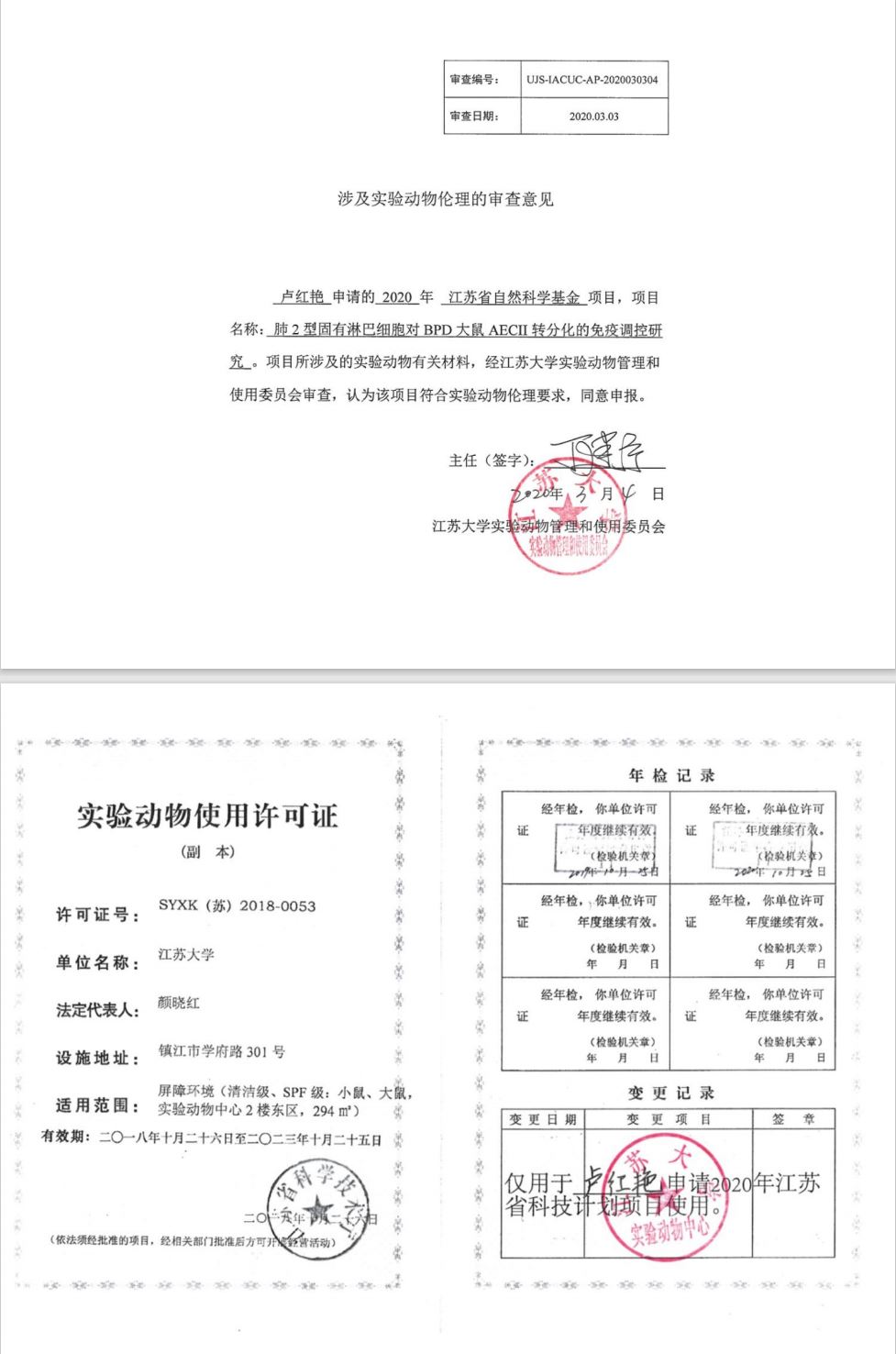


**Supplementary Figure 5.**  **Institutional Review Board Statement**

The study was reported in accordance with ARRIVE guidelines and approved by the Animal Center at Jiangsu University (protocol No. UJS-IACUC-AP-2020030304 and approved on 2020.03.03)

**3 SUPPLEMENTARY FIGURE**

**Supplementary Table S1**:

Table S1 Primer pairs used for RT-qPCR.

| Gene |  | Primer Sequence (5'-3') |
| --- | --- | --- |
| MR | F | 5′-caaggaaggttggcatttgt-3′ |
|  | R | 5′-cctttcagtcctttgcaagc-3′ |
| Arginase | F | 5′-agtctggcagttggaagcat-3′ |
|  | R | 5′-ctggttgtcaggggagtgtt-3′ |
| HO-1 | F | 5′-ggtgatggcttccttgtacc-3′ |
|  | R | 5′-agtgaggcccataccagaag-3′ |
| Fizz1 | F | 5′-tgctgggatgactgctactg-3′ |
|  | R | 5′-ctgggttctccacctcttca-3′ |
| IL-10 | F | 5′-ccagtacagccgggaagaca-3′ |
| IL-33  ST2 | R  F  R  F  R | 5′-cagctggtcctttgtttgaaaga-3′  5′-ctactgcatgagactccgttctg-3′  5′-tgtgtgagggacactccttac-3′  5′-tcgaaatgaaagttccagca-3′  5′-tgtgtgagggacactccttac-3′ |

**Supplementary Table S2**:

Table S2 List of Abbreviation.

| Abbreviation | Definition |
| --- | --- |
| AM | alveolar macrophage |
| M1 | classically activated macrophage |
| M2 | alternatively activated macrophage |
| BPD | bronchopulmonary dysplasia |
| AECII | Type II alveolar epithelial cell |
| AECI | type I alveolar epithelial cell |
| EMT | epithelial-mesenchymal transition |
| MLI | mean linear intercept |
| MAA | mean alveolar area |
